# Lactic acid-containing products for bacterial vaginosis and their impact on the vaginal microbiota: A systematic review

**DOI:** 10.1371/journal.pone.0246953

**Published:** 2021-02-11

**Authors:** Erica L. Plummer, Catriona S. Bradshaw, Michelle Doyle, Christopher K. Fairley, Gerald L. Murray, Deborah Bateson, Lindi Masson, Josephine Slifirski, Gilda Tachedjian, Lenka A. Vodstrcil

**Affiliations:** 1 Central Clinical School, Monash University, Melbourne, Victoria, Australia; 2 Melbourne Sexual Health Centre, Alfred Hospital, Carlton, Victoria, Australia; 3 Women’s Centre for Infectious Diseases, The Royal Women’s Hospital, Parkville, Victoria, Australia; 4 Murdoch Children’s Research Institute, Parkville, Victoria, Australia; 5 Department of Obstetrics and Gynaecology, The University of Melbourne, Parkville, Victoria, Australia; 6 Family Planning New South Wales, Ashfield, New South Wales, Australia; 7 Discipline of Obstetrics, Gynaecology and Neonatology, University of Sydney, Camperdown, New South Wales, Australia; 8 Burnet Institute, Melbourne, Victoria, Australia; 9 Division of Medical Virology, Department of Pathology, University of Cape Town, Cape Town, South Africa; 10 Institute of Infectious Disease and Molecular Medicine (IDM), University of Cape Town, Cape Town, South Africa; 11 Centre for the AIDS Programme of Research in South Africa, Durban, South Africa; 12 Department of Microbiology, Monash University, Clayton, Victoria, Australia; 13 Department of Microbiology and Immunology, University of Melbourne, at the Peter Doherty Institute of Infection and Immunity, Melbourne, Victoria, Australia; University of Maryland School of Medicine, UNITED STATES

## Abstract

**Objective:**

The vaginal microbiota in bacterial vaginosis (BV) typically has low abundance of lactic acid producing lactobacilli. Lactic acid has properties that may make it effective for treating BV and/or restoring an optimal lactobacillus-dominated vaginal microbiota. We conducted a systematic review to describe the effect of intravaginal lactic acid-containing products on BV cure, and their impact on vaginal microbiota composition (PROSPERO registration: CRD42018115982).

**Methods:**

PubMed, Embase and OVID were searched from inception to November 2019 to identify eligible studies. Included studies evaluated an intravaginal lactic acid-containing product and reported BV cure using established diagnostic methods, and/or vaginal microbiota composition using molecular methods. Studies were independently screened and assessed, and the proportion of women cured post-treatment was calculated. Study results were described in a qualitative manner.

**Results:**

We identified 1,883 articles and assessed 57 full-texts for eligibility. Seven different lactic acid-containing products were evaluated and differed with respect to excipients, lactic acid concentration and pH. Most studies had medium or high risk of bias. Three trials compared the efficacy of a lactic acid-containing product to metronidazole for BV cure. One study found lactic acid to be equivalent to metronidazole and two studies found lactic acid to be significantly inferior to metronidazole. Two studies included a control group receiving a placebo or no treatment. One reported lactic acid to be superior than no treatment and the other reported lactic acid to be equivalent to placebo. Lactic acid-containing products did not significantly impact the vaginal microbiota composition.

**Conclusion:**

There is a lack of high-quality evidence to support the use of lactic acid-containing products for BV cure or vaginal microbiota modulation. However, adequately powered and rigorous randomised trials with accompanying vaginal microbiota data are needed to evaluate the efficacy of lactic acid as a BV treatment strategy.

## Introduction

Bacterial vaginosis (BV) is the commonest vaginal condition in reproductive aged women. BV is associated with serious sequelae including miscarriage, preterm birth and pelvic inflammatory disease, and acquisition of sexually transmitted infections including HIV [1–5]. Recommended first-line treatments for BV are oral or intravaginal metronidazole and intravaginal clindamycin [6]. First-line treatments have equivalent four-week cure rates of ~70–85% [7], but BV recurrence is common [8, 9]. Recurrences negatively impact a woman’s quality of life [10] and result in repeated clinical presentations and antibiotic use. Given the significant sequelae, treatments that improve BV cure are needed.

The optimal vaginal microbiota of reproductive aged women is typically characterised by dominance of lactic acid producing *Lactobacillus* species including *Lactobacillus crispatus*, *Lactobacillus gasseri* and *Lactobacillus jensenii* [11–16]. Women with BV have reduced abundance of these lactobacilli and increased prevalence and abundance of anaerobic and facultative-anaerobic bacteria [13, 14]. *In vitro* studies have shown that lactic acid inactivates BV-associated bacteria [17] and pathogens including *Chlamydia trachomatis*, *Neisseria gonorrhoeae* and HIV via mechanisms independent of acidity alone [18–21]. Lactic acid has also been shown to modulate cervicovaginal epithelial cell functions to prevent *C*. *trachomatis* infection [22]. Lactic acid also has immunomodulatory effects [23], and can elicit an anti-inflammatory response and reduce production of inflammatory cytokines and chemokines from cervicovaginal epithelial cells *in vitro* [24].

The antimicrobial and immunomodulatory properties of lactic acid may make it effective for the treatment of BV and/or to restore an optimal microbiota following antibiotic treatment [23]. Lactic acid-containing products have been evaluated for BV treatment in clinical trials, and several over-the-counter lactic acid-containing products are marketed to treat BV or support optimal vaginal microbiota. However, the use of these products is not recommended by any treatment guidelines [6].

We conducted a systematic review with two objectives: 1) to describe the effect of intravaginal lactic acid-containing products for BV cure (assessed using an established diagnostic method), and 2) to describe the impact of intravaginal lactic acid-containing products on the vaginal microbiota (assessed using molecular methods).

## Materials and methods

We conducted and reported this systematic review according to the Preferred Reporting Items for Systematic Reviews and Meta-Analysis statement [25] (S1 File), and registered the protocol prospectively with PROSPERO (CRD42018115982).

### Search strategy, eligibility criteria

We searched electronic databases (PubMed, Embase, OVID Medline) from inception until 4th November 2019 using keywords: “bacterial vaginosis”, “vaginal microbiota” and “lactic acid” (search strings in S1 Table). Reference lists and conference abstracts were searched for additional studies. Conference abstracts were included if they reported adequate information. Studies were uploaded to Covidence (Veritas Health Innovation, Melbourne, Australia, www.covidence.org) and were independently reviewed for eligibility by three authors (EP, JS, MD). Disagreements were resolved with a fourth author (LV).

Studies were eligible for objective 1 (BV cure outcome) if they assessed an intravaginal lactic acid-containing product as the main or adjuvant therapy for BV cure in women diagnosed with BV. BV had to be diagnosed using an established method (e.g. Amsel criteria or modified Amsel criteria [26], Nugent Score [NS] [27] or Ison-Hay method [28]). Studies were eligible if they were randomised controlled trials (RCT) where an intravaginal lactic acid-containing product was assessed in comparison to either no treatment, a placebo or a recommended antibiotic treatment for BV. No restrictions were placed on number of participants enrolled. Studies of pregnant women and post-menopausal women were excluded.

Studies were eligible for objective 2 (vaginal microbiota outcome) if they reported use of an intravaginal lactic acid-containing product in women with or without BV, and assessed the vaginal microbiota using a molecular method such as quantitative PCR (qPCR) or high throughput sequencing. In order to capture all published literature evaluating the impact of lactic acid-containing product on the vaginal microbiota composition, no restrictions were placed on study design, number of participants enrolled, age, menopause or pregnancy status.

For both objectives, we excluded studies if they were performed on animals or the data was not stratified by lactic acid-containing product use. Only English language studies were included.

### Interventions assessed

Assessed interventions included any intravaginal lactic acid-containing product. Interventions were excluded if they contained lactic acid producing bacteria or were not used intravaginally.

### Outcome measures

Outcome measures were: 1) BV cure defined as ≤2 Amsel criteria and/or NS<4, or Ison-Hay grade 1 measured ≥7 days after the start of treatment, 2) vaginal microbiota composition assessed by molecular methods, and 3) occurrence of adverse events.

### Data extraction

Three authors (EP, JS, MD) independently extracted the following information for each study: author details, publication year, study design, population studied, intervention details, comparator details, follow-up duration, BV diagnostic method, BV cure definition, microbiota characterisation methodology, adverse events and study findings. Disagreements in extracted data were resolved by discussion between authors. Two corresponding authors were contacted for additional details, one responded [29].

### Data analysis

For objective 1 (BV cure outcome), we calculated the proportion of women cured post-treatment per treatment group with 95% confidence intervals, and described results in a narrative manner. For objective 2 (vaginal microbiota outcome), the impact of lactic acid-containing products on the vaginal microbiota was described narratively. Where an article reported ≥2 lactic acid-containing products or treatment regimens, each product/regimen was presented separately in tables.

### Assessment of bias

Two authors (EP, MD) independently assessed the risk of bias of each study using a modified version of the RoB 2.0 [30] and ROBINS-I tools [31] (S2 Table). The level of overall risk was summarised across six domains: selection bias, performance bias, measurement bias, response bias, reporting bias and other sources of bias (i.e. adjustment for confounders and insufficient description of product details). Studies were not excluded based on bias assessment.

## Results

### Study selection

**Fig 1:**
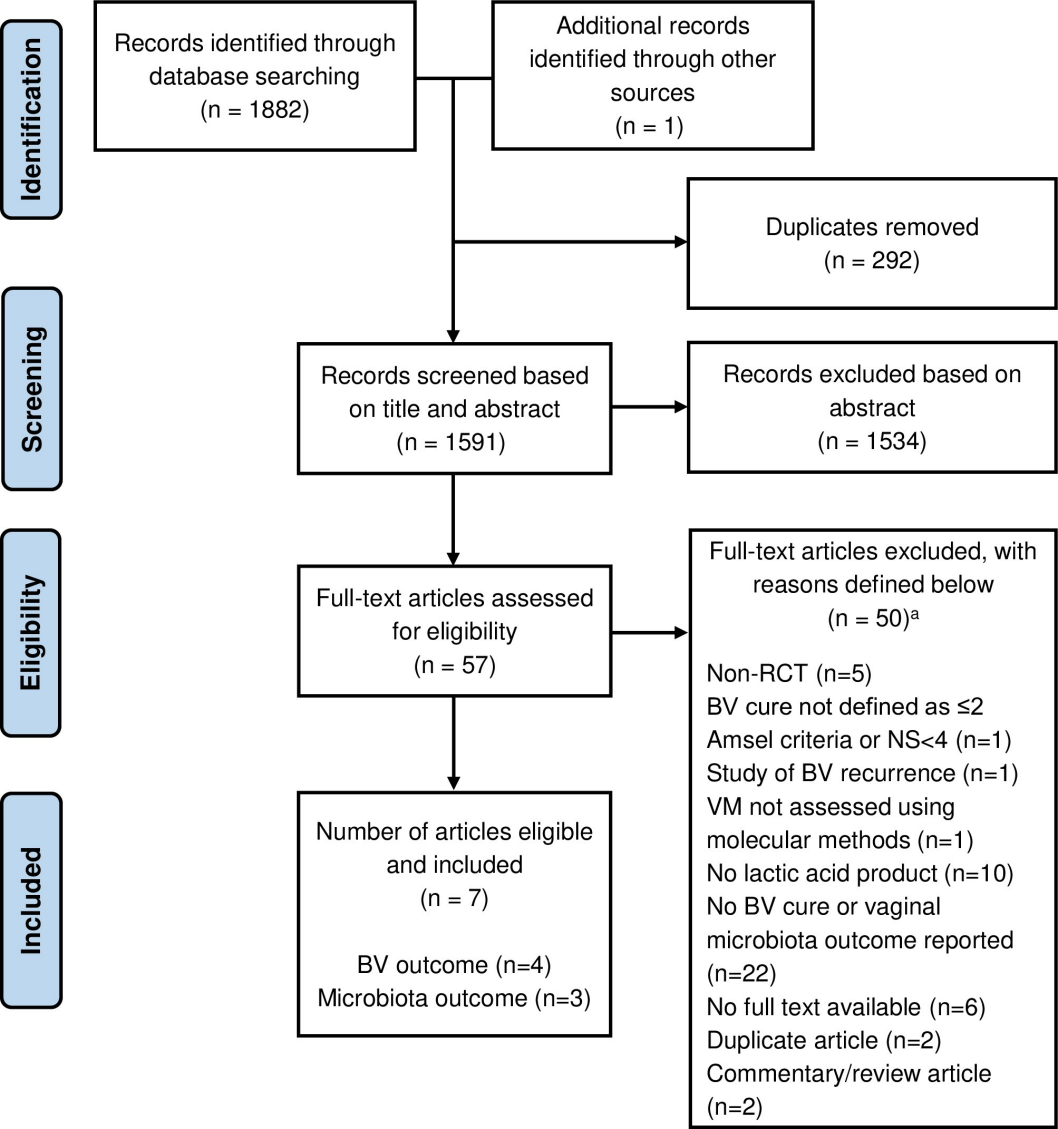
PRISMA flow diagram describing the literature search and article selection process. ^a^ Detailed reasons for exclusion are provided in S3 Table.

Our initial search identified 1882 articles. One additional article was identified from reference lists. Following duplicate removal, 1591 articles were screened on title and abstract. We excluded 1534 articles and assessed 57 full-text articles. Fifty articles were excluded; seven of which evaluated a lactic acid-containing product for BV treatment but were excluded because they were non-randomised (n = 5), did not use standard criteria to assess BV cure (n = 1) or assessed BV-recurrence only (n = 1; S3 Table). Seven articles were included in the review (Fig 1).

### Lactic acid-containing products evaluated

Seven different lactic acid-containing products were evaluated and differed with respect to lactic acid concentration, pH and included ingredients/excipients (Table 1). Two intravaginal gels were evaluated in three studies: Acidform was evaluated in two studies [32, 33] and Lactal was evaluated in one study [34]. Four different vaginal pessaries were evaluated in three studies [29, 35, 36] and a vaginal douche (Etos®) was evaluated in one study [37]. Excipients were not reported in one study [36]. Lactic acid isomer details were only located for Acidform, which comprises L-lactic acid [38].

**Table 1:** Lactic acid-containing product details.

Product name, formulation, reference	Lactic acid details	pH	Other ingredients and excipients including preservatives
Acidform^a^ intravaginal gel [32, 33]	88mg (1.76%) per dose	3.55	50 mg (1%) citric acid, 20 mg (0.4%) potassium bitartrate, benzoic acid, alginic acid, xanthan gum, glycerin, sodium hydroxide and water in a 5mg dose [38]
	L-lactic acid isomer		
Lactal intravaginal gel [34] ^b^	NR	3.5	Growth substrates for lactobacilli
Lactic acid pessary [35]	100mg lactic acid per pessary	3.3	2.4g of polyethylene glycol 1540
Vagisan®, vaginal pessary [29]	40mg lactic acid per pessary	~4.5	Macrogol 1500, macrogol 6000 and sodium lactate
WO3191, vaginal pessary [29]	Total lactic acid conc. of ~3.9% total weight	~4.5	Cocoamphopropionate (amphoteric tenside), sodium lactate
Sustained release oligomeric lactic acid (OMLA) pessary [36]	700mg lactic acid per pessary^c^	pH 3.5	NR
Etos® vaginal douche [37]	Neat lactic acid conc. 0.45%, diluted conc.	Neat pH 3.42, diluted pH 3.50 (1 in 7 dilution)	Aqua, butylene glycol, caprylyl glycol, sodium pyroglutamic acid, Zea mays kernel extract, hydrolyzed milk protein, niacinamide, and adenosine triphosphate
	0.06% (1 in 7 dilution)		

NR, not reported.

^a^ Also known as Amphora.

^b^ Reference [39] states that Lactal gel is the lactic acid-containing product in Andersch *et al*. [34].

^c^ Designed to release lactic acid over a 72hr period.

### Intravaginal lactic acid-containing products for BV cure

Four RCTs assessed the efficacy of an intravaginal lactic acid-containing product for BV cure (Table 2) [32, 34–36].

**Table 2:** Key findings of included studies.

**Objective 1: Studies assessing BV cure**							
**Reference**	**Study design**	**Intervention**	**Comparator**	**No. women randomised**	**Outcome measure**	**Duration of follow-up**	**BV cure results in intervention vs comparator**
Andersch, 1986 [34]	RCT^a^	Lactal gel 5ml PV/night x 7 nights	Oral MTZ 500 mg bid x 7 days	Lactal = 32	≤2 of 3 Amsel criteria^b^	1 week after start of treatment	31/31 (100%, 95%CI 89–100) *vs*
				MTZ = 22			17/17 (100%, 95%CI 90–100)
Boeke, 1993 [35]	RCT^a^	Oral placebo bid x 7 days and lactic acid vaginal suppository/night x 7 days	Two comparator groups:	Lactic acid = 41^c^	≤2 of 4 Amsel criteria	2 weeks after start of treatment	18/37 (49%, 95%CI 32–66) *vs*
				MTZ = 44			35/42 (83%, 95%CI 69–93)
			1) Oral MTZ 500 mg bid x 7 days and placebo vaginal suppository/night x 7 days	Placebo = 40			16/34 (47%, 95%CI 30–65)
						4 weeks after start of treatment	11/33 (33%, 95%CI 18–52) *vs*
							27/38 (71%, 95%CI 54–85)
			2) Oral placebo bid x 7 days and placebo vaginal suppository/night x 7 days				12/35 (34%, 95%CI 19–52)
						3 months after start of treatment	12/32 (38%, 95%CI 21–56) *vs*
							29/37 (78%, 95%CI 62–90)
							11/32 (34%, 95%CI 19–53)
Simoes, 2006 [32]	Double-blind RCT	Acidform gel 5g PV/day x 5 days	10% MTZ gel PV/day x 5 days	Acidform = 13	≤2 of 4 Amsel criteria	12–17 days after start of treatment	3/13 (23%, 95%CI 5–54) *vs*
				MTZ = 17			15/17 (88%, 95%CI 64–99)
						33–40 days after start of treatment	1/13 (8%, 95%CI 0–36) *vs*
							9/17 (53%, 95%CI 28–77)
Fredstorp, 2015 [36]	Open-label RCT	Two intervention groups:	Untreated control group	Once/week = 37	≤2 of 4 Amsel criteria	1 week after start of treatment	24/34 (71%, 95%CI 53–85)
				Twice/week = 35			28/35 (80%, 95%CI 63–92) *vs*
		1) OMLA pessary applied once/week for 1 week		Control = 33			3/30 (10%, 95%CI 2–27)
		2) OMLA pessary applied twice/week for 1 week^d^					
**Objective 2: Studies assessing vaginal microbiota composition**							
**Reference**	**Study design**	**Intervention**	**Comparator**	**No. women randomised**	**Outcome measure**	**Reported results**	
Keller, 2012 [33]	Single-blind RCT	Acidform gel 5g PV bid x 14 days	HEC placebo gel PV bid x 14 days	Acidform = 18	qPCR assays:	In 35^e^ women without BV, no significant changes were observed in the prevalence or concentration of *L*. *crispatus*, *L*. *jensenii*, *Megasphaera* (type 1 & type 2) or BVAB2 following 14 days of gel use in either the Acidform or placebo group (compared to baseline values).	
				Placebo = 18	*L*. *crispatus*		
					*L*. *jensenii*		
					*G*. *vaginalis*		
					*Megasphaera* (type 1 & type 2)		
						There was a non-significant trend towards a decrease in *G*. *vaginalis* concentration in the Acidform group following 14 days of gel use compared to baseline (median of 1.36x106 to 3.66x104 DNA copies/swab, p = 0.083), but not in the placebo group (median of 9.8x105 to 4.4x106 DNA copies/swab, p-value not reported).	
					BVAB2		
Gottschick, 2017 [29] ^f^	Double-blind RCT	Oral MTZ 2g single dose. After 7–28 days, WO3191 pessary applied twice-weekly x 3 weeks	Oral MTZ 2g single dose. After 7–28 days, Vagisan® pessary applied twice-weekly x 3 weeks	WO3191 = 18	16S rRNA gene sequencing of V1-V2 regions	In 36^g^ women initially treated for BV with oral metronidazole, no significant changes in vaginal microbiota composition were reported during or following use of either WO3191 or Vagisan®.	
				Vagisan® = 26			
						The cumulative relative abundance of *Lactobacillus* spp. (*L*. *crispatus*, *L*. *iners* and *L*. *gasseri*) was 73% in the WO3191 group prior to starting pessary use, 77% after 3 weeks of pessary use, and had decreased to 59% 12–14 weeks after last pessary use.	
						The cumulative relative abundance of *Lactobacillus* spp. was 75% in the Vagisan® group prior to starting pessary use, 69% after 3 weeks of pessary use, and 73% 12–14 weeks after last pessary use. There was a non-significant increase in the relative abundance of *L*. *crispatus* in Vagisan® group from 18% prior to starting pessary use to 33% 12–14 weeks after last pessary use.	
						There was no difference in microbiota diversity (as measured by Shannon diversity index) between women randomised to WO3191 and women randomised to Vagisan®.	
van der Veer, 2019 [37]	Single arm prospective cohort	Participants were followed for 3 menstrual cycles. Etos® douche was applied 3/per week for duration of cycle 2 starting on day 1 of menses.	NA	29	16S rRNA gene sequencing of V3-V4 regions	In 25^h^ women without BV there was a non-statistically significant increased odds of having a diverse anaerobic vaginal microbiota relative to an *L*. *crispatus* microbiota during (odds ratio: 1.4; 95% CI 0.9–2.1) and after douching with Etos® (odds ratio: 1.7; 95%CI 0.9–3.1), compared to before douching, following adjustment for menses.	
						Douching with Etos® had no effect on microbiota diversity as measured by Shannon diversity index.	

No., number; BV, bacterial vaginosis; RCT, randomised controlled trial; PV, intravaginal; MTZ, metronidazole; bid, twice a day; OMLA, oligometric lactic acid; CI, confidence interval; qPCR, quantitative PCR; NA, not applicable.

^a^ Details of blinding not provided.

^b^ The three criteria evaluated were: positive amine test, clue cells, pH≥5.0.

^c^ 168 women randomised, but post-randomisation, 43 women were found to be ineligible and excluded, thus randomisation numbers presented reflect the 125 eligible women included in analyses.

^d^ OMLA pessary is designed to release lactic acid over a 72hr period.

^e^ One woman allocated to Acidform did not receive the intervention.

^f^ Both the intervention (WO3191) and the comparator (Vagisan®) contain lactic acid.

^g^ 36 women were included in microbiota analyses, n = 13 receiving WO3191 and n = 23 receiving Vagisan®.

^h^ Twenty-nine women were recruited, four were excluded and 25 women completed the study.

**Fig 2:**
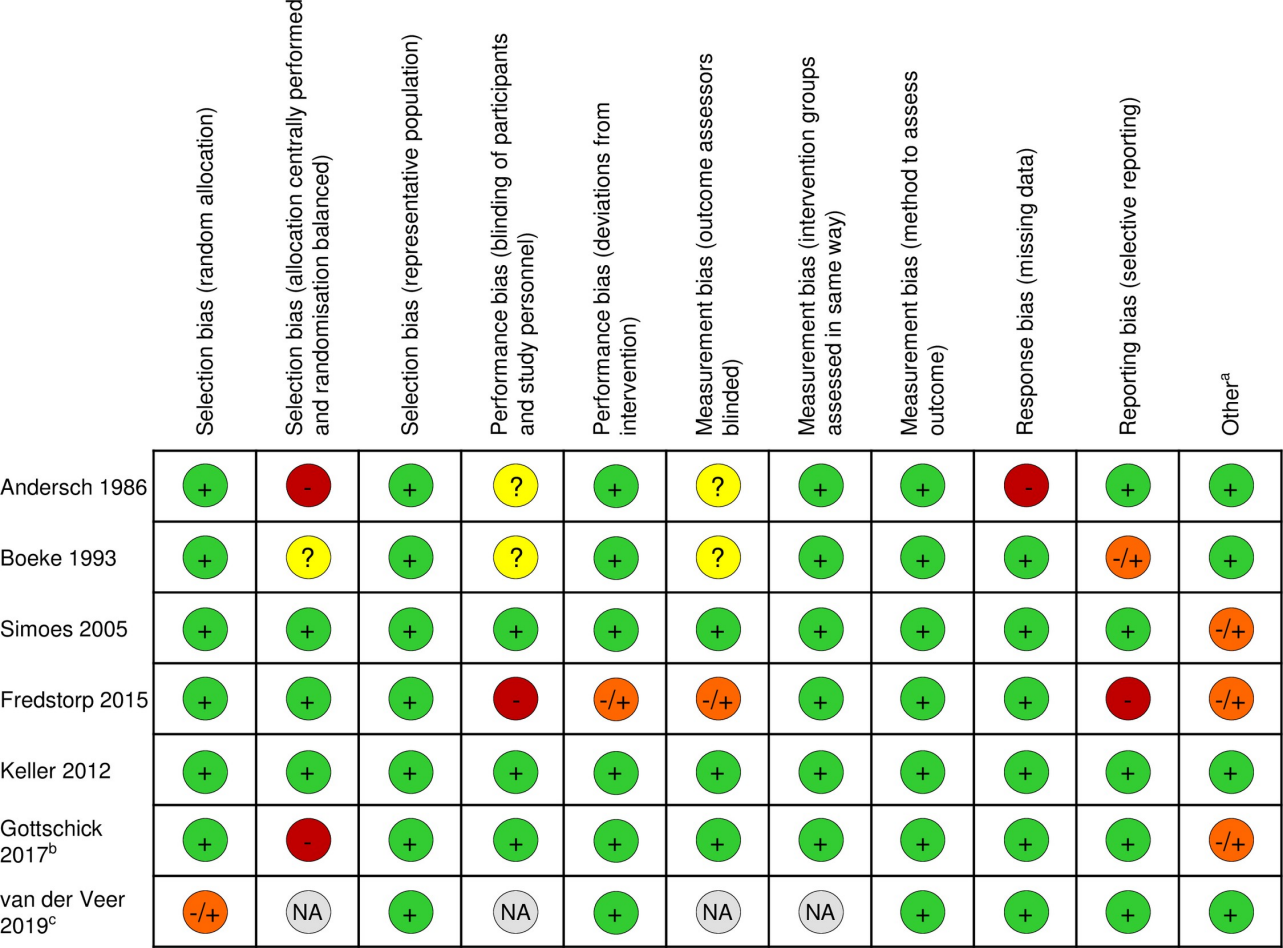
Risk of bias assessment. + indicates a low risk of bias, -/+ indicates moderate risk of bias,—indicates high risk of bias,? indicates unknown risk, NA indicates bias is not applicable to the study. ^a^ Other sources of bias include whether confounders were appropriately accounted for and whether lactic acid-containing product details were sufficiently described in the manuscript. ^b^ One study disclosed receipt of funding from the lactic acid-containing product manufacturer. ^c^ Single arm prospective cohort study.

Andersch *et al*. [34] randomised women to receive once nightly Lactal gel (lactic acid concentration not specified) for seven days or twice daily (*bid*) oral metronidazole for seven days. No details of allocation concealment, implementation of randomisation or blinding of participants and/or Amsel outcome assessors were provided (Fig 2). One week after the start of treatment (i.e. immediately post-treatment), all women in both groups had ≤2 of 3 Amsel criteria (Table 2); 77% (n = 24/31) of women receiving Lactal and 76% (n = 13/17) of women receiving metronidazole were negative for all criteria assessed (positive amine test, clue cells, pH≥5.0). No adverse events were reported (S4 Table).

In a multicentre RCT, Boeke *et al*. [35] randomised women to receive either nightly lactic acid pessary (100mg lactic acid/pessary) plus oral placebo *bid* for seven days, oral metronidazole *bid* plus nightly placebo pessary for seven days, or oral placebo *bid* plus nightly placebo pessary for seven days. No details of allocation concealment or blinding were provided (Fig 2). Cure was assessed at three time points (2-weeks, 4-weeks and 3-months after the start of treatment) using ≤2 of 4 Amsel criteria and an additional author definition called ‘strict’ cure (absence of: positive amine test, clue cells, pH>4.5). Two weeks after the start of treatment, 49% (n = 18/37) of women receiving lactic acid, 83% (n = 35/42) of women receiving metronidazole and 47% (n = 16/34) of women receiving dual placebo had ≤2 Amsel criteria (Table 2). When assessed according to the authors definition of strict cure, none of the women receiving lactic acid pessary, 10% of women receiving metronidazole and 3% of women receiving dual placebo were cured. Similar findings were reported 4 weeks and 3-months after start of treatment (Table 2). There was no difference in adverse events reported between the three randomisation groups. Of 33 women receiving lactic acid, four reported gastrointestinal symptoms, three reported genital irritation and one reported headache/vertigo (S4 Table).

In a double-blind pilot RCT, Simoes *et al*. [32] randomised women to receive either once daily Acidform gel (an acid buffering contraceptive gel, 88mg lactic acid/5g) for five days (n = 13) or once daily 10% metronidazole intravaginal gel for five days (n = 17). Randomisation was performed by the product manufacturer and researchers were provided with product tubes labelled with participant numbers so that the randomisation group was double-blinded to the researchers and participants (Fig 2). At 7–12 days post-treatment, 23% (n = 3/13) of women receiving Acidform and 88% (n = 15/17) women receiving metronidazole were cured (≤2 Amsel criteria). At 28–35 days post-treatment, the percent of women cured decreased to 8% (n = 1/13) in the Acidform group and 53% (n = 9/17) in the metronidazole group (Table 2). Four women receiving Acidform and one woman receiving metronidazole reported genital irritation (S4 Table).

Fredstorp *et al*. [36] evaluated a sustained release oligomeric lactic acid (OMLA) pessary in a two part multicentre study. Part A of the study is not included in this review as there was no control group. In Part B, women were randomised to receive either OMLA pessary applied once per week for one week, OMLA pessary applied twice per week for a week or no treatment. Block randomisation was performed according to a computer-generated randomisation list, with block size blinded to the investigators. Sites were provided with coded envelopes, and the study was open-label (Fig 2). After one week of pessary use, 71% (n = 24/34) of women receiving once-weekly pessary, 80% (n = 28/35) of women receiving twice-weekly pessary and 10% (n = 3/30) of women receiving no treatment had ≤2 of 3 Amsel criteria (Table 2). Vaginal itching was the most common adverse event and was reported by 11 women receiving OMLA pessary, by five applying the pessary once/week and by six applying the pessary twice/week. Two women receiving OMLA pessary had a yeast infection, and vaginal irritation and genital burning sensation were both reported by >1 woman (exact numbers and group not provided; S4 Table). Adverse events were not recorded from control participants.

### Impact of intravaginal lactic acid-containing products on the vaginal microbiota composition

Three studies reported a measure of vaginal microbiota composition (Table 2) [29, 33, 37].

Keller *et al*. [33] evaluated the safety of Acidform gel *bid* (88mg lactic acid/5g) compared to HEC placebo gel *bid* in 35 sexually abstinent non-pregnant women without BV. Women were randomised 1:1 by a pharmacist. Though the treatments were not identical in appearance, participants were not informed of their allocation and laboratory personnel assessing the outcome were blinded (Fig 2). The change in prevalence and concentration of five bacteria after 14 days of gel use was assessed by qPCR. There were no significant changes in vaginal microbiota composition following 14 days of either Acidform or placebo (Table 2). A non-significant trend towards decreased *Gardnerella vaginalis* concentration following Acidform use was reported. Five women receiving Acidform reported vulvar itching, four reported vaginal or vulvar burning and three reported abdominal cramping (S4 Table). Two women receiving placebo reported vaginal or vulvar itching.

Gottschick *et al*. [29] evaluated the safety, tolerability and efficacy of a biofilm disrupting agent (cocoamphopropionate) administered as a pessary (WO3191, which contains lactic acid at 3.9% of total pessary weight) in a double-blind RCT. Forty-four non-pregnant women were randomised to receive either WO3191 or Vagisan® (40mg lactic acid/pessary) 7–28 days after treatment for BV with 2g single dose oral metronidazole (Table 2). No details of randomisation or allocation concealment were provided (Fig 2). Microbiota results (assessed by 16S rRNA gene sequencing) were reported for 36 women (WO3191 n = 13 and Vagisan® n = 23). No significant changes in vaginal microbiota composition were observed during or following use of either pessary. No safety concerns were identified for either pessary (S4 Table).

In an open-label non-comparative pilot study, van de Veer *et al*. [37] evaluated the impact of a lactic acid-containing douche (Etos®, 0.06% lactic acid when diluted for use) on the vaginal microbiota composition of 25 non-pregnant reproductive aged women without BV (Table 2). Etos® did not significantly impact the vaginal microbiota composition (assessed by 16S rRNA gene sequencing). The study reported non-significant increased odds for having a diverse anaerobic vaginal microbiota during and after douching with Etos®, following adjustment for menses. Five women reported dryness and 2 reported an increase in vaginal symptoms post douching (S4 Table).

### Adverse events

No major safety concerns were reported (S4 Table). Vaginal or vulvar irritation, itching, burning, redness and/or dryness were recorded in women receiving a lactic acid-containing product in five of the seven studies. Minimal differences in adverse events between lactic acid-containing product and control randomisation groups were reported.

### Risk of bias of included studies

Risk of bias assessment is in Fig 2. Only one RCT evaluating a lactic acid-containing product for BV cure was double-blinded [32] and only one study had low bias across all six domains [33].

Two studies assessing BV cure reported sample size calculations [35, 36] and one reached the required sample size [36]. Four studies measured treatment adherence; one study reported these results [33]. An additional study reported comparable treatment adherence across intervention groups, but did not provide raw data [35].

## Discussion

The efficacy of lactic acid-containing products for BV cure and their impact on the vaginal microbiota composition has not been extensively evaluated. We identified four RCTs that investigated the use of intravaginal lactic acid-containing products for BV cure and three studies that investigated the impact of lactic acid-containing products on the vaginal microbiota. Most studies were small and underpowered, had medium-high risk of bias, and the time-point at which cure was measured differed between studies. Three studies compared a lactic acid-containing product to a first-line BV treatment: one reported lactic acid to have equivalent efficacy to metronidazole and two reported lactic acid to be inferior to metronidazole. Two studies included a placebo or no treatment control group: one reported lactic acid to be superior to no treatment and the other reported lactic acid to be equivalent to placebo. Minimal effects of lactic acid-containing products on the vaginal microbiota were reported. New treatments are needed to improve BV cure and the use of lactic acid is supported by *in vitro* evidence [17, 23]. However, there is limited high-quality *in vivo* evidence that supports the use of lactic acid for BV cure or modulating the vaginal microbiota. Large rigorous trials of well evaluated lactic acid-containing products with long-term follow-up and accompanying microbiota data are needed.

The lactic acid-containing products assessed varied with respect to lactic acid concentration, pH, formulation (i.e. gel, pessary, douche) and excipients. Women with lactobacillus-dominated vaginal microbiota (defined as NS = 0–3) have an average vaginal lactic acid concentration of approximately 0.79–1% and pH of 3.45–4.12 [40, 41]. Some products had a lactic acid concentration or pH outside of these ranges, and no study reported the concentration of lactic acid released into the vagina. Thus, it is not clear if biologically active levels of lactic acid were achieved, which may have impacted on treatment efficacy. Functional effects of lactic acid *in vitro* are usually observed within concentration ranges of 0.30–1% and pH of 3.45–4.12 [18, 40, 41], and are mediated by the uncharged protonated form of lactic acid which predominates at pH≤3.86 [17, 18, 24]. Accordingly, biological effects diminish as lactic acid levels decrease and pH increases. For example, at <0.3% lactic acid and pH≥4.2, the HIV virucidal activity [18] and immunomodulatory effects [24] of lactic acid decrease. Additionally, while 1% lactic acid at pH 4.5 reduces the viability of BV-associated bacteria approximately 10^6^-fold, a negligible reduction is observed with 0.1% lactic acid [17]. The lactic acid concentration and vaginal pH maintained after dosing are likely to be critical for achieving biological effects *in vivo*.

Other important characteristics of lactic acid-containing products need consideration, including lactic acid isomer and product osmolality. Lactic acid exists in two isomers: D- and L-lactic acid, and *Lactobacillus* spp. differ in their ability to produce each isomer. For example, *in vitro*, *L*. *crispatus* and *L*. *gasseri* produce both isomers, *L*. *jensenii* produces only D-lactic acid and *L*. *iners* produces only L-lactic acid [42]. The protective effects of *L*. *crispatus* compared to *L*. *iners* are partly attributed to the ability of *L*. *crispatus* to produce D-lactic acid [23]. It is hypothesised that D-lactic acid affords more protection than L-lactic acid against upper genital tract infections [42]; however, this has not been studied in the context of BV. Isomer information was available for one product included in this review. In order to understand the relative contribution of each isomer to the inactivation of BV-associated bacteria, future studies of products under evaluation for BV treatment or vaginal microbiota modulation should report the L‐/D‐isomer ratio. Additionally, no study reported product osmolality. This is relevant because hyperosmolal products are likely to damage vaginal epithelium [43, 44]. Vaginal and vulvar irritation were commonly reported adverse events in women using lactic acid-containing products, and may be related to product osmolality and/or excipients or other ingredients (e.g. citric acid). Adverse events should be monitored following intravaginal lactic acid use.

Minimal changes in vaginal microbiota composition following lactic acid-containing product use were reported. Two of the three studies evaluating microbiota composition recruited women without BV and the third study assessed women recently treated with oral metronidazole. Thus, one might expect the impact of lactic acid on the vaginal microbiota composition of these women to be minimal. The non-significant association of Etos® douche with non-optimal vaginal microbiota composition [45] may be a result of the douching action rather than an adverse impact of lactic acid, highlighting the importance of product formulation. Douching has been associated with increased risk of BV-associated bacteria detection [46], as well as increased risk of intermediate-BV and Nugent-BV by meta-analysis [4]. However, whether douching introduces BV-associated bacteria, depletes optimal lactobacilli, or modifies the vaginal environment such that BV-associated bacteria growth is favoured is unknown.

This review has limitations. The 2019 Food and Drug Administration (FDA) guidelines for developing BV treatments recommends that clinical cure be defined as the absence of 3 Amsel criteria, specifically resolution of vaginal discharge, a negative whiff test and clue cells <20% per high-power field on wet mount [47]. In clinical practice, BV is typically diagnosed as the presence of ≥3 Amsel criteria [6], recurrence is defined as the presence of ≥3 criteria [48] and cure is reported as ≤2 criteria. Based on international clinical practice and published studies, we defined BV cure as the presence of ≤2 Amsel criteria (and/or NS<4, although no included study reported cure using NS) and not by the 2019 FDA guidelines. Additionally, only two studies assessed cure at a timepoint recommended by FDA guidelines [32, 35]. The FDA guidelines recommend cure be assessed 7–14 days post-randomisation for topical drugs administered for a short period of time (i.e. 1–2 days) or 21–30 days post-randomisation for topical drugs that that are administered for a longer period of time (i.e. 1 week) [47]. Follow-up was limited to immediately post-treatment in two studies [34, 36], which is not only likely to be too soon after treatment cessation to adequately assess cure, it also prevented our assessment of the long-term efficacy and safety of lactic acid-containing products. If lactic acid is effective it is likely to be most effective when used as adjunctive therapy with antibiotics [23] and/or when used as sustained release or as periodic presumptive therapy, as has been shown with biweekly suppressive use of 0.75% metronidazole gel [49]. Finally, our search was restricted to English-language records which excluded at least one study [50].

Other lactic acid-containing products are available over-the-counter but were either not identified through our systematic search of published literature or were ineligible for inclusion in our review. An RCT of 1,900 women comparing the clinical and cost effectiveness of intravaginal lactic acid gel to oral metronidazole for BV is currently ongoing [51] (ISRCTN14161293). The primary outcome is patient reported resolution of BV symptoms 14-days post-randomisation. Initial qualitative data from ISRCTN14161293 indicates women prefer lactic acid gel to antibiotics for mild BV episodes despite lower perceived efficacy [52], supporting the need to further investigate lactic acid-containing products for BV.

## Conclusions

New treatments are needed to improve BV cure, reduce associated sequelae and improve antibiotic stewardship. *In vitro* data suggest that lactic acid may be effective for BV treatment; however, high-quality evidence supporting the use of lactic acid-containing products for BV and modification of the vaginal microbiota is lacking. Large, rigorous randomised trials of lactic acid-containing products that have been carefully evaluated with respect to pH, lactic acid concentration, L‐/D‐isomer ratio and osmolality are needed. Future studies should include standardised clinical endpoints, standardised timing of endpoint measurement, assessment of adverse events, long-term follow-up of participants and accompanying high-resolution vaginal microbiota data.

## Supporting information

S1 File PRISMA checklist. (DOC)

S1 Table Database search strings. (DOCX)

S2 Table Bias assessment tool. (DOCX)

S3 Table Full text articles excluded and reasons for exclusion. (DOCX)

S4 Table Adverse events reported in included studies. (DOCX)
