# Supplementary material for: Lactic acid-containing products for bacterial vaginosis and their impact on the vaginal microbiota: A systematic review
Source: PLoS One. 2021 Feb 11;16(2):e0246953. doi: 10.1371/journal.pone.0246953 (PMC7877752; doi:10.1371/journal.pone.0246953)
Supplement: S1 Table — (DOCX) [file pone.0246953.s002.docx]

**S1 Table. Database search strings**

| **Database** | **Search string** | **Date of most recent search** |
| --- | --- | --- |
| PubMed | ((((bacterial AND vagin* OR vaginosis OR vaginitis OR (vagin* AND infect*) OR gardnerella OR (vagina* AND bacteri*) OR (vagina* AND microbio*) OR (vagina* AND flora) OR (cervicovagina* AND flora) OR (cervicovagina* AND microbio*)))) AND ((lact* AND acid OR lactic OR lactate))) AND english[Language] | 4-Nov-19 |
| OVID Medline | (((bacterial and vagin*) or vaginosis or vaginitis or (vagin* and infect*) or gardnerella or (vagina* and bacteri*) or (vagina* and microbio*) or (vagina* and flora) or (cervicovagina* and flora) or (cervicovagina* and microbio*)) and ((lact* and acid) or lactic or lactate)).af. and English.lg. | 4-Nov-19 |
| Embase | (bacterial AND vagin* OR vaginosis OR vaginitis OR (vagin* AND infect*) OR gardnerella OR (vagina* AND bacteri*) OR (vagina* AND microbio*) OR (vagina* AND flora) OR (cervicovagina* AND flora) OR (cervicovagina* AND microbio*)) AND (lact* AND acid OR lactic OR lactate) AND 'english':la | 4-Nov-19 |
