# Supplementary material for: Lactic acid-containing products for bacterial vaginosis and their impact on the vaginal microbiota: A systematic review
Source: PLoS One. 2021 Feb 11;16(2):e0246953. doi: 10.1371/journal.pone.0246953 (PMC7877752; doi:10.1371/journal.pone.0246953)
Supplement: S2 Table — (DOCX) [file pone.0246953.s003.docx]

**S2 Table. Bias assessment tool**

| **First author name and year:** | | | | **Reviewer name:** |
| --- | --- | --- | --- | --- |
| **What outcome/s does study contribute to? BV, microbiota or both:** | | | |  |
|  | **Bias item** | **Level of risk of bias (low, moderate, high or unclear)** | **Risk judgement** | **Support for judgement** |
|  |  |  |  |  |
| **Selection bias.** | Were participants randomly allocated? If not randomised, were they sequentially enrolled? | **Yes (low risk):** patients randomly allocated treatment | **+** |  |
|  |  | **No (moderate risk):** not randomised, but sequentially enrolled | **-/+** |  |
|  |  | **No (high risk):** patients self-selected their treatment or were given treatment without any listing/explanation of eligibility criteria | **-** |  |
|  |  | **Could not be determined (unknown risk)** | **?** |  |
|  | Allocation concealment. Was allocation performed centrally e.g. (phone, web, pharmacy), and was allocation concealed? | **Yes (low risk):** allocation performed centrally using predetermined random sequence. e.g. Sequentially numbered sealed envelopes, identical drug containers e | **+** |  |
|  |  | **No (high risk):** allocation not performed centrally, allocation sequence was predictable or known to staff. | **-** |  |
|  |  | **Could not be determined (unknown risk)** | **?** |  |
|  | Was the recruited population representative of the general population? | **Yes (low risk):** population was clearly representative | **+** |  |
|  |  | **No (high risk):** population was clearly not representative i.e. pregnant women, sex workers etc | **-** |  |
|  |  | **Could not be determined (unknown risk)** | **?** |  |
| **Performance bias** | Were study participants and personnel blinded to what intervention a participant received, and was blinding effective? | **Yes (low risk):** participants and personnel blinded, blinding was effective and unlikely that blinding could have been broken | **+** |  |
|  |  | **No (moderate risk):** no blinding but outcome unlikely to be influenced | **-/+** |  |
|  |  | **No (high risk):** no blinding or incomplete or broken blinding, outcome likely to be influenced | **-** |  |
|  |  | **Could not be determined (unknown risk)** | **?** |  |
|  | Were there deviations from the intended intervention beyond what would be expected in usual practice? If so, were deviations likely to affect the outcome? | **No (Low risk):** there were no deviations from the intended intervention | **+** |  |
|  |  | **Yes (moderate risk):** the study deviated from the intended intervention but the deviations were unlikely to have affected the outcome | **-/+** |  |
|  |  | **Yes (high risk):** the study deviated from the intended intervention and the deviations were likely to have affected the outcome | **-** |  |
|  |  | **Could not be determined (unknown risk)** | **?** |  |
| **Measurement bias** | **BV outcome -** Were the assessors of the outcome blinded to the intervention? | **Yes (low risk):** assessors were blinded to the intervention and it is unlikely that blinding could have been broken | **+** |  |
|  |  | **No (moderate risk):** no blinding but measurement of outcome unlikely to be influenced | **-/+** |  |
|  |  | **No (high risk):** no blinding or incomplete or broken blinding, measurement of outcome likely to be influenced. | **-** |  |
|  |  | **Could not be determined (unknown risk)** | **?** |  |
|  | **BV outcome -** Were the intervention groups assessed in the same way? | **Yes (low risk):** the intervention groups were assessed in the same way | **+** |  |
|  |  | **No (high risk):** the intervention groups were not assessed in the same way | **-** |  |
|  |  | **Could not be determined (unknown risk)** | **?** |  |
|  | **BV outcome -** Methodology used to assess outcome | **Low risk:** ≥3 Amsel’s criteria and/or Nugent Score: 7-10 | **+** |  |
|  |  | **Moderate risk:** <3 Amsel’s criteria i.e. clue cells, discharge, pH or amine/odour | **-/+** |  |
|  |  | **Could not be determined (unknown risk)** | **?** |  |
|  | **Microbiota outcome** - Were the assessors of the outcome blinded to the intervention? | **Yes (low risk):** assessors of the outcome were blinded to the intervention and it is unlikely that blinding could have been broken. | **+** |  |
|  |  | **No (moderate risk):** no blinding of assessors of the outcome but measurement of outcome unlikely to be influenced | **-/+** |  |
|  |  | **No (high risk):** no blinding or incomplete or broken blinding of assessors of the outcome, measurement of outcome likely to be influenced. | **-** |  |
|  |  | **Could not be determined (unknown risk)** | **?** |  |
|  | **Microbiota outcome** - Were the intervention groups assessed in the same way? | **Yes (low risk):** the intervention groups were assessed in the same way | **+** |  |
|  |  | **No (high risk):** the intervention groups were not assessed in the same way | **-** |  |
|  |  | **Could not be determined (unknown risk)** | **?** |  |
|  | **Microbiota outcome** - Methodology used to assess outcome | **Low risk:** 16S rRNA gene sequencing or qPCR assays | **+** |  |
|  |  | **Moderate risk:** Nugent score only | **-/+** |  |
|  |  | **Could not be determined (unknown risk)** | **?** |  |
| **Response bias** | Assessment of missing data | **Low risk:** no missing data, OR data is missing but reasons for missing are not related to outcome, missing data is balanced across groups, proportion of missing data is not enough to have significant impact on outcome | **+** |  |
|  |  | **High risk:** data is missing and reasons for missing data are not provided or related to outcome or imbalanced between groups. Large enough numbers of missing data to have an impact on results. | **-** |  |
|  |  | **Could not be determined (unknown risk)** | **?** |  |
| **Reporting bias** | Was selective reporting performed? | **No (low risk):** all pre-specified outcomes relevant to the review were reported in the pre-specified way | **+** |  |
|  |  | **Yes (high risk):** outcomes are not reported as expected or as pre-specified in protocol/paper | **-** |  |
|  |  | **Could not be determined (unknown risk)** | **?** |  |
| **Other** | Was confounding accounted for appropriately? (applicable to non RCTs) | **Yes (low risk):** confounding variables taken into account in the design and/or analysis (e.g. through matching, stratification, interaction terms, multivariate analysis, or other statistical adjustment) | **+** |  |
|  |  | **No (High risk):** no adjustment or consideration of confounding variables | **-** |  |
|  |  | **Could not be determined (unknown risk)** | **?** |  |
|  | Are the lactic acid treatment details sufficiently described in the manuscript? | **Yes (low risk):** manuscript clearly details formulation and mode of administration of lactic acid treatment | **+** |  |
|  |  | **No (moderate risk):** details of lactic acid treatment are missing from the manuscript, however drug name provided so details can be obtained from other sources | **-/+** |  |
|  |  | **No (high risk):** no details of lactic acid treatment formulation in manuscript. | **-** |  |
|  |  | **Could not be determined (unknown risk)** | **?** |  |

Developed using the RoB 2.0 (*Revised* Cochrane *risk of bias tool* for *randomized trials*) and ROBINS-I (Risk Of Bias In Non-randomised Studies - of Interventions) tools. + indicates a low risk of bias, -/+ indicates moderate risk of bias, - indicates high risk of bias, ? indicates unknown risk.
