# Supplementary material for: Lactic acid-containing products for bacterial vaginosis and their impact on the vaginal microbiota: A systematic review
Source: PLoS One. 2021 Feb 11;16(2):e0246953. doi: 10.1371/journal.pone.0246953 (PMC7877752; doi:10.1371/journal.pone.0246953)
Supplement: S3 Table — (DOCX) [file pone.0246953.s004.docx]

**S3 Table. Full text articles excluded and reasons for exclusion**

| **Reference** | **Reason/s for exclusion** |
| --- | --- |
| Rapisarda AMC, Caldaci L, Valenti G, Brescia R, Sapia F, Sarpietro G, et al. Efficacy of vaginal preparation containing *Lactobacillus acidophilus*, lactic acid and deodorized garlic extract in treatment and prevention of symptomatic bacterial vaginitis: result from a single-arm pilot study. Italian Journal of Gynaecology & Obstetrics. 2018;30(1):21-31. | Non-RCT Contains a lactic acid producing probiotic |
| Hirnle L, Malolepsza-Jarmolowska K, Kubis AA, Hirnle P. Evaluation of bacterial vaginosis therapy in pregnant women with vaginal tablets containing lactic acid complexed with eudragit® E-100 which undergo gelation at the site of application. Advances in Clinical and Experimental Medicine. 2006;15(4):645-51. | Non-RCT Pregnant women Definition of BV at baseline is unclear and difficult to determiune the number of women with NS=7-10 at enrolment who were cured (NS<4) at endpoint |
| Holst E, Brandberg A. Treatment of bacterial vaginosis in pregnancy with a lactate gel. Scand J Infect Dis. 1990;22(5):625-6. | Non-RCT Pregnant women |
| Bahamondes MV, Portugal PM, Brolazo EM, Simoes JA, Bahamondes L. Use of a lactic acid plus lactoserum intimate liquid soap for external hygiene in the prevention of bacterial vaginosis recurrence after metronidazole oral treatment. Rev Assoc Med Bras (1992). 2011;57(4):415-20. | Non-RCT Study of BV recurrence Product used externally |
| Di Pierro F, Catacchio V, Candidi C, Zerbinati N, Alfonso R. Rhatany-based preparation in vulvovaginitis and vaginosis. Gazzetta Medica Italiana Archivio per le Scienze Mediche. 2009;168(6):339-46. | Non-RCT BV cure not defined as ≤2 Amsel criteria and/or NS<4 Contains a lactic acid producing probiotic |
| Decena DC, Co JT, Manalastas RM, Jr., Palaypayon EP, Padolina CS, Sison JM, et al. Metronidazole with Lactacyd vaginal gel in bacterial vaginosis. J Obstet Gynaecol Res. 2006;32(2):243-51. | BV cure not defined as ≤2 Amsel criteria and/or NS<4 |
| Andersch B, Lindell D, Dahlen I, Brandberg A. Bacterial vaginosis and the effect of intermittent prophylactic treatment with an acid lactate gel. Gynecol Obstet Invest. 1990;30(2):114-9. | Study of BV recurrence |
| Amaral E, Perdigao A, Souza MH, Mauck C, Waller D, Zaneveld L, et al. Vaginal safety after use of a bioadhesive, acid-buffering, microbicidal contraceptive gel (ACIDFORM) and a 2% nonoxynol-9 product. Contraception. 2006;73(5):542-7. | Study of women without BV where vaginal microbiota is not assessed using molecular methods (Assessed only by Nugent Score) |
| Andersch B, Lindell D, Dahlen I, Brandberg A. Bacterial vaginosis and the effect of intermittent prophylactic treatment with an acid lactate gel. Gynecol Obstet Invest. 1990;30(2):114-9. | Duplicate record |
| Boeke AJ, Dekker JH, van Eijk JT, Kostense PJ, Bezemer PD. Effect of lactic acid suppositories compared with oral metronidazole and placebo in bacterial vaginosis: a randomised clinical trial. Genitourinary medicine. 1993;69(5):388-92 | Duplicate record |
| Brittingham A, Wilson WA. The antimicrobial effect of boric acid on trichomonas vaginalis. Sexually Transmitted Diseases. 2014;41(12):718-22. | No BV cure or vaginal microbiota outcome |
| Carati D, Zizza A, Guido M, De Donno A, Stefanizzi R, Serra R, et al. Safety, efficacy, and tolerability of differential treatment to prevent and treat vaginal dryness and vulvovaginitis in diabetic women. Clinical and experimental obstetrics & gynecology. 2016;43(2):198-202. | No BV cure or vaginal microbiota outcome |
| Clackson TE, Coombs GH. The antagonistic effects of acetate and lactate upon the trichomonacidal activity of metronidazole. Journal of Antimicrobial Chemotherapy. 1983;11(5):401-6. | No BV cure or vaginal microbiota outcome |
| Eusaph AZ, Nighat R, Arshad A. Lactacyd FH as an adjuvant therapy for vulvovaginal infections in Pakistani women: Fresh study, a satisfaction survey. Journal of the Pakistan Medical Association. 2016;66(5):521-7. | No BV cure or vaginal microbiota outcome |
| Guaraldi C, Costantino M, Costantino D. Tyndallized lactic ferments: New possible therapies in treating vaginitis. Minerva Ginecologica. 2017;69(1):112-5. | No BV cure or vaginal microbiota outcome |
| Jones CP, Carter B, Thomas WL. The treatment of resistant or recurrent vaginal trichomoniasis with lactic acid jelly and lactic acid douches. 1960;149(Suppl):128-38. | No BV cure or vaginal microbiota outcome |
| Kale V, Patil M, Khandagade A. Development of vaginal tablets containing probiotic and prebiotic. International Journal of Pharmaceutical Sciences Review and Research. 2012;15(1):31-5. | No BV cure or vaginal microbiota outcome |
| Lee YK, Chung HH, Kim JW, Park NH, Song YS, Kang SB. Vaginal pH-balanced gel for the control of atrophic vaginitis among breast cancer survivors: A randomized controlled trial. Obstetrics and Gynecology. 2011;117(4):922-7. | No BV cure or vaginal microbiota outcome |
| Malolepsza-Jarmolowska K. Studies on gynaecological hydrophilic lactic acid preparations, Part 7: use of chitosan as lactic acid carrier in intravaginal tablets (globuli vaginales). Die Pharmazie. 2006;61(9):780-2. | No BV cure or vaginal microbiota outcome |
| Malolepsza-Jarmolowska K. Studies on gynecological hydrophilic lactic acid preparations. Part 8: use of chitosan as lactic acid carrier in intravaginal tablets. Acta poloniae pharmaceutica. 2007;64(1):69-72. | No BV cure or vaginal microbiota outcome |
| Malolepsza-Jarmolowska K, Kubis AA, Hirnle L. Studies on gynaecological hydrophilic lactic acid preparations. Part 5: The use of Eudragit E-100 as lactic acid carrier in intravaginal tablets. Die Pharmazie. 2003;58(4):260-2. | No BV cure or vaginal microbiota outcome |
| Malolepsza-Jarmolowska K, Kubis AA, Hirnle L. Studies on gynaecological hydrophilic lactic acid preparations, part 6: use of Eudragit E-100 as lactic acid carrier in intravaginal tablets. Die Pharmazie. 2003;58(5):334-6. | No BV cure or vaginal microbiota outcome |
| Mauck CK, Brache V, Kimble T, Thurman A, Cochon L, Littlefield S, et al. A phase I randomized postcoital testing and safety study of the Caya diaphragm used with 3% Nonoxynol-9 gel, ContraGel or no gel. Contraception. 2017;96(2):124-30. | No BV cure or vaginal microbiota outcome |
| Melvin L, Glasier A, Elton R, Cameron ST. pH-balanced tampons: Do they effectively control vaginal pH? BJOG: An International Journal of Obstetrics and Gynaecology. 2008;115(5):639-45. | No BV cure or vaginal microbiota outcome |
| Passloer HJ. Problems of povidone-iodine and LEC as an alternative in vaginal disinfection. International Journal of Experimental and Clinical Chemotherapy. 1990;3(4):235-7. | No BV cure or vaginal microbiota outcome |
| Sanchez Carazo JL, Gimeno Carpio E, Grifol ALA. Comparison of three imidazolic regimens in the treatment of vaginal candidosis. European Journal of Sexually Transmitted Diseases. 1986;3(4):223-5. | No BV cure or vaginal microbiota outcome |
| Swidsinski A, Loening-Baucke V, Mendling W, Swidsinski S. Positive effects of local therapy with a vaginal lactic acid gel on dysuria and E.coli bacteriuria question our current views on recurrent cystitis. Archives of Gynecology and Obstetrics. 2012;285(6):1619-25. | No BV cure or vaginal microbiota outcome |
| Tansupasiri A, Puangsricharern A, Itti-arwachakul A, Asavapiriyanont S. Satisfaction and tolerability of combination of lactoserum and lactic acid on the external genitalia in Thai women. Journal of the Medical Association of Thailand. 2005;88(12):1753-7. | No BV cure or vaginal microbiota outcome |
| Tedeschi C, Benvenuti C. Comparison of vaginal gel isoflavones versus no topical treatment in vaginal dystrophy: results of a preliminary prospective study. Gynecol Endocrinol. 2012;28(8):652-4. | No BV cure or vaginal microbiota outcome |
| Verstraelen H, Vervaet C, Remon J-P. Rationale and Safety Assessment of a Novel Intravaginal Drug-Delivery System with Sustained DL-Lactic Acid Release, Intended for Long-Term Protection of the Vaginal Microbiome. PLoS ONE. 2016;11(4):e0153441. | No BV cure or vaginal microbiota outcome |
| Weissenbacher ER, Schulze K. Tampovagan®-C lactic acid - Investigation of the galenic preparation and clinical and microbiological efficacy in non-specific vaginitis. International Journal of Feto-Maternal Medicine. 1991;4(2):79-86. | No BV cure or vaginal microbiota outcome |
| Brzezinski A, Stern T, Arbel R, Rahav G, Benita S. Efficacy of a novel pH-buffering tampon in preserving the acidic vaginal pH during menstruation. International Journal of Gynecology and Obstetrics. 2004;85(3):298-300. | No BV cure or vaginal microbiota outcome |
| Lamotte C, Neut C, Decrocq N, Djotni H. Saforelle® and the vulvovaginal ecosystem. Fundamental and Clinical Pharmacology. 2011;25:53 | No full text available |
| Maneksha S. Comparison of povidone iodine (betadine) vaginal pessaries and lactic acid pessaries in the treatment of vaginitis. Journal of International Medical Research. 1974;2(3):236-9. | No full text available |
| Tedeschi C, Benvenuti C. Use of topical+oral isoflavones in vaginal dystrophy. Climacteric. 2011;14:196-7. | No full text available |
| Piot P, Van Dyck E, Godts P, Vanderheyden J. A placebo-controlled, double-blind comparison of tinidazole and triple sulfonamide cream for the treatment of nonspecific vaginitis. American Journal of Obstetrics and Gynecology. 1983;147(1):85-9. | No full text available |
| Thomas M, Culwell K, Howard B, Dart C. Gynecologic infections and colposcopy findings from a phase 3 efficacy and safety study of a contraceptive vaginal gel compared with nonoxynol-9. Contraception. 2016;94(4):415. | No full text available |
| Viravaidya S, Manonai J, Sarit-Apirak S, Wattanayingcharoenchai R. Effects of topical antiseptic agent on vaginal symptoms, ph and infection in postmenopausal women using pessary for pelvic organ prolapse. International Urogynecology Journal and Pelvic Floor Dysfunction. 2012;23(2):S131-S2. | No full text available |
| Fleury F, Hodgson C. Single-dose treatment of vulvovaginal candidiasis with a new 500 mg clotrimazole vaginal tablet. Advances in Therapy. 1984;1(5):349-56. | No lactic acid therapy |
| Ahmad FJ, Alam MA, Khan ZI, Khar RK, Ali M. Development and in vitro evaluation of an acid buffering bioadhesive vaginal gel for mixed vaginal infections. Acta Pharmaceutica. 2008;58(4):407-19. | No lactic acid therapy |
| Alam MA, Ahmad FJ, Khan ZI, Khar RK, Ali M. Development and evaluation of acid-buffering bioadhesive vaginal tablet for mixed vaginal infections. AAPS PharmSciTech. 2007;8(4):E109. | No lactic acid therapy |
| Alioua S, Abdi A, Fhoula I, Bringel F, Boudabous A, Ouzari I. Diversity of vaginal lactic acid bacterial microbiota in 15 Algerian pregnant women with and without bacterial vaginosis by using culture independent method. Journal of Clinical and Diagnostic Research. 2016;10(9):DC23-DC7. | No lactic acid therapy |
| Apolikhina IA, Sukhih GT, Teterina TA, Aslanyan KO, Kuzmin SG, Vorozhtsov GN. Antiviral and antimycotic effects of PDT with ALAsense. Photodiagnosis and Photodynamic Therapy. 2011;8(2):170-1. | No lactic acid therapy |
| Holloway P, Bojovic T, Bojovic D, Bontekoe R, Boon T, Schuren F, et al. A reduction in gardnerella vaginales and bacteroides in cervical samples of women treated for bacterial vaginosis following treatment with a topical mucoadhesive gel. Cytopathology. 2011;22:79. | No lactic acid therapy |
| Minis EE, Moron A, Forney L, Leizer J, Bongiovanni AM, Linhares IM, et al. Second trimester D-lactic acid measurement: A simple assay to predict dominant vaginal bacteria and risk for short cervix and preterm birth. American Journal of Obstetrics and Gynecology. 2018;218(1):S421-S2. | No lactic acid therapy |
| Nyirjesy P, Robinson J, Mathew L, Lev-Sagie A, Reyes I, Culhane JF. Alternative therapies in women with chronic vaginitis. Obstetrics and Gynecology. 2011;117(4):856-61. | No lactic acid therapy |
| Ozmen S, Turhan NO, Seckin NC. Gardnerella-associated vaginitis: Comparison of three treatment modalities. Turkish Journal of Medical Sciences. 1998;28(2):171-3. | No lactic acid therapy |
| Quaranta L, Ottolina J, Parma M, Chionna R, Sileo F, Dindelli M, et al. An alternative approach for the treatment of vaginal atrophy. Minerva Ginecologica. 2014;66(4):377-81. | No lactic acid therapy |
| Davis S. Vaginal pH-balanced gel for the control of atrophic vaginitis in breast cancer survivors. Climacteric. 2011;14(4):507-8. | Commentary /Review article |
| Aldunate M, Srbinovski D, Hearps AC, Latham CF, Ramsland PA, Gugasyan R, et al. Antimicrobial and immune modulatory effects of lactic acid and short chain fatty acids produced by vaginal microbiota associated with eubiosis and bacterial vaginosis. Front Physiol. 2015;6:164. | Commentary /Review article |
