# Supplementary material for: Lactic acid-containing products for bacterial vaginosis and their impact on the vaginal microbiota: A systematic review
Source: PLoS One. 2021 Feb 11;16(2):e0246953. doi: 10.1371/journal.pone.0246953 (PMC7877752; doi:10.1371/journal.pone.0246953)
Supplement: S4 Table — (DOCX) [file pone.0246953.s005.docx]

**S4 Table. Adverse events reported in included studies**

| **Study** | **Lactic acid-containing product** | **Summary of adverse events in women receiving lactic acid-containing product** | **Summary of adverse in control group where applicable** |
| --- | --- | --- | --- |
| Andersch et al, 1986 | Lactal | No women reported side effects | 30% of women receiving MTZ reported mild side effects including nausea, metallic taste or gastrointestinal discomfort |
| Boeke et al, 1993 | Lactic acid pessary | Most participants (76%) reported no side effects. 12% of women receiving lactic acid pessary reported gastrointestinal side effects, 9% reported vaginal side effects and 1 woman reported headache. No women receiving lactic acid had *Candida ablicans* detected. | Most participants receiving MTZ (80%) reported no side effects. 7% of women reported gastrointestinal side effects, 8% reported vaginal side effects, 6% reported bad taste or other adverse event and 13% had *C. albicans* detected.  Most participants receiving placebo (80%) reported no side effects. 11% of women reported gastrointestinal side effects, 3% reported vaginal side effects, 3% reported headache headache/vertigo, 6% reported other adverse events and 9% had *C. albicans* detected |
| Simoes et al, 2005 | Acidform | Genital irritation was reported by 4/13 women receiving Acidform. 7/13 women were Candida positive (4/13 had symptomatic Candida infection) | Genital irritation was reported by 1/17 women receiving MTZ. 7/17 women were Candida positive (3/17 had symptomatic Candida infection) |
| Keller et al, 2012 | Acidform | 65% of women who received Acidform had at least one local adverse event. Commonly reported side effects were vulvar itching, vaginal or vulvar burning and abdominal cramping. | Two women receiving placebo reported vaignal or vulvar itching. |
| Fredstorp et al, 2015 | Sustained release oligomeric lactic acid (OMLA) pessary | The most common side-effects were vaginal itching (mentioned by <20% of participants) and/or vaginal burning. Two women developed a yeast infection. | NA - Adverse events were not reported for women in the control group. |
| Gottschick et al, 2017 | WO3191 pessary and Vagisan® pessary | No safety concerns identified with either WO3191 or Vagisan®. Tolerability of the products was judged as being good/very good in most women. The most commonly reported adverse event was bacterial vaginosis, which was comparable in both groups. | NA |
| van der Veer et al, 2019 | Etos® vaginal douche | Five women reported dryness and 2 reported an increase in vaginal symptoms post douching.  Women were three-fold more likely to test positive for *Candida* at the end of cycle 2 (menstrual cycle when women douched with Etos), compared to cycle 1 (before douching) [OR = 3.0 (95% CI: 1.2–7.2); p = 0.017] | NA |

NA, not applicable; MTZ, metronidazole.
